# Effects of pentacene-doped PEDOT:PSS as a hole-conducting layer on the performance characteristics of polymer photovoltaic cells

**DOI:** 10.1186/1556-276X-7-5

**Published:** 2012-01-05

**Authors:** Hyunsoo Kim, Jungrae Lee, Sunseong Ok, Youngson Choe

**Affiliations:** 1 Department of Chemical Engineering, Pusan National University, Busan, 609-735, South Korea

**Keywords:** electronic materials, polymers, vapor deposition, electrochemical measurement, electrochemical properties

## Abstract

We have investigated the effect of pentacene-doped poly(3,4-ethylenedioxythiophene:poly(4-styrenesulfonate) [PEDOT:PSS] films as a hole-conducting layer on the performance of polymer photovoltaic cells. By increasing the amount of pentacene and the annealing temperature of pentacene-doped PEDOT:PSS layer, the changes of performance characteristics were evaluated. Pentacene-doped PEDOT:PSS thin films were prepared by dissolving pentacene in 1-methyl-2-pyrrolidinone solvent and mixing with PEDOT:PSS. As the amount of pentacene in the PEDOT:PSS solution was increased, UV-visible transmittance also increased dramatically. By increasing the amount of pentacene in PEDOT:PSS films, dramatic decreases in both the work function and surface resistance were observed. However, the work function and surface resistance began to sharply increase above the doping amount of pentacene at 7.7 and 9.9 mg, respectively. As the annealing temperature was increased, the surface roughness of pentacene-doped PEDOT:PSS films also increased, leading to the formation of PEDOT:PSS aggregates. The films of pentacene-doped PEDOT:PSS were characterized by AFM, SEM, UV-visible transmittance, surface analyzer, surface resistance, and photovoltaic response analysis.

## Background

Recently, among the photovoltaic cells considered as renewable energy sources, organic photovoltaic cells such as nanoscale polymer semiconductors have been intensively developed [1]. As alternative technologies to conventional photovoltaic cells, polymer bulk-heterojunction [BHJ] photovoltaic cells have gained great attention since they have several advantages such as low-cost fabrication, mechanical flexibility [2,3], and easy fabrication process including spin-coating [4]. The BHJ-structured device is an intimate blend of donor and acceptor materials that are phase-separated into nanodomains, where one or both materials absorb photons to form bound electron-hole pairs (excitons). An interpenetrating network in the BHJ structure provides a large interfacial area for efficient exciton dissociation [5,6], leading to high efficiency of device performance.

Poly(3,4-ethylenedioxythiophene:poly(4-styrenesulfonate) [PEDOT:PSS] is the most widely utilized polymer as a hole-conducting layer of OLED and photovoltaic cells [7]. The advantages of PEDOT:PSS include low temperature, excellent stability, large area processing, low cost, and flexibility. However, the efficiency of this material is limited by its low carrier mobility [8]. Therefore, hole mobility is a key parameter for photovoltaic devices with respect to their adoption in device applications. Pentacene has been extensively studied as a p-type semiconductor in organic field-effect transistors, and the field-effect hole mobility of pentacene is reported to be about 1.5 cm^2^/Vs [9,10]. In addition, pentacene has long exciton diffusion length and well-suited absorption spectrum. Because the advantages offered by pentacene are attributed to a good semiconducting behavior, many theoretical and experimental studies were focused on its crystal structure, morphology, optical, and electrical transport properties [11-13]. Many researchers have reported on photovoltaic applications of pentacene as a dopant into a hole-conducting layer [14,15], an interlayer for polymer BHJ photovoltaic cells and a donor material [16]. Surface morphology, work function, and transmittance of the pentacene-doped PEDOT:PSS films improve a high hole mobility and conductivity.

In this study, poly(3-hexylthiophene-2,5-diyl) [P3HT] and [6,6]-phenyl-C_61_-butyric acid methyl ester [PCBM] were blended and used as an active layer in polymer BHJ photovoltaic cells. The performance characteristics of polymer photovoltaic cells using pentacene-doped PEDOT:PSS as a hole-conducting layer have been investigated. In details, an investigation is taken to understand the effect of pentacene-doped PEDOT:PSS films on the performance of polymer photovoltaic cells with various amounts of pentacene in a PEDOT:PSS solution. We present the fabrication of efficient polymer photovoltaic cells by optimizing the parameters including the amount of pentacene and annealing temperature of pentacene-doped PEDOT:PSS thin films, which are important parameters because these can affect power conversion efficiency.

## Methods

### Materials

Indium tin oxide [ITO] thin films were used as the anode because they combine unique transparency and conducting properties. They have a wide bandgap (3.8 eV) and show high transmission in the visible wavelength (80 ~ 90%) and relatively high work function. The ITO glass substrates were supplied from Samsung Corning Precision Materials Co., Ltd. (Gumi-si, South Korea). PEDOT:PSS aqueous solution (Baytron P VP A14083;1.3 wt.%) as a buffer-layer material was purchased from H. C. Starck (Goslar, Germany). 1-Methyl-2-pyrrolidinine [NMP] as a solvent, pentacene as a doping material, and 1,2-dichlorobenzene as a solvent were purchased from Sigma-Aldrich (Seoul, South Korea). P3HT as an electron donor was purchased from Rieke Metal Inc. (Lincoln, NE, USA). PCBM as an electron acceptor was purchased from Nano-C (Westwood, MA, USA). Aluminum as a cathode was purchased from CERAC™, Inc. (Milwaukee, WI, USA).

### Device fabrication

The pre-patterned ITO glass substrates were cleaned with acetone, ethanol, and isopropyl alcohol (1:1:1) for 1 h by sonication and then rinsed by ethanol. After cleaning, the ITO glass substrates were annealed at 230°C for 10 min in vacuum and served as high-work-function electrode. PEDOT:PSS and pentacene were used as buffer-layer materials. Various amounts of pentacene (1.3, 3.3, 5.5, 7.7, and 9.9 mg) were dissolved in 3.2 g of NMP solvent. The color of the pentacene solution became dark purple and slowly turned into intense yellow as the dissolution time increased. The PEDOT:PSS solution was filtered using a 0.45-μm PTFE syringe filter (Millipore, Seoul, South Korea), and then the pentacene solution was mixed with 3.2 g of PEDOT:PSS. PEDOT:PSS solutions containing pentacene were stirred for 1 h and then spin-coated on the ITO substrate at 2,000 RPM for 20 s using a digitalized spin coater (MS-A10, Mikasa Co., Ltd., Minato-ku, Tokyo, Japan). The pentacene-doped PEDOT:PSS thin films were annealed for 1 h at 120°C, 140°C, 160°C, and 180°C in vacuum to remove the aqueous PSS. After the annealing process, the devices were cooled down to room temperature. The typical thickness of the pentacene-doped PEDOT:PSS thin film was about 40 nm in this work.

The BHJ of the active-layer thin film was prepared via a solution process. P3HT and PCBM were dissolved into 1,2-dichlorobenzene in a weight ratio of 1:0.9 and various concentrations of 2.0 wt.% solution. The blend of P3HT and PCBM was stirred for 24 h at 40°C. The blend of the P3HT:PCBM solution was spin-casted on the pentacene-doped PEDOT:PSS buffer layer at 1,000 RPM for 40 s. The thickness of the P3HT:PCBM blend's thin film is about 450 nm. After the spin-coating, to form the active layer, a cathode electrode, Al, was deposited onto the active layer by thermal evaporation in vacuum with a thickness of 100 nm. The thickness was measured using a well-calibrated quartz crystal thickness monitor (CRTM-600, ULVAC KIKO Co., Ltd., Yokohama-shi, Kanggawa, Japan). The vacuum pressure was under 3 × 10^-5 ^torr, and the deposition rate of aluminum was controlled at 1 ~ 5 Å/s. The fabricated devices were subsequently post-annealed for 10 min at 150°C in vacuum condition.

## Results and discussion

**Figure 1:**
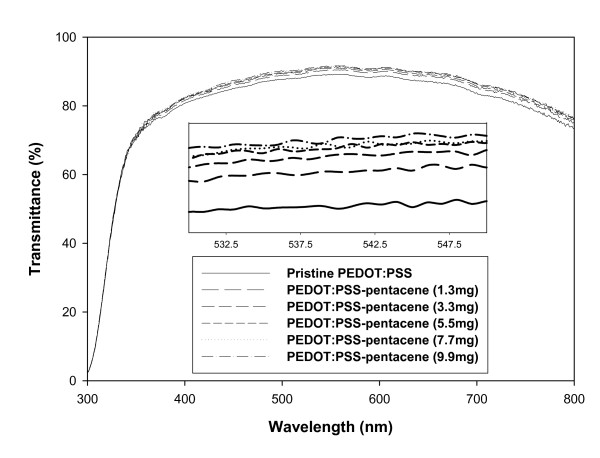
**UV-visible transmittance spectra of pentacene-doped PEDOT:PSS films**. The inset shows the magnified spectra from 530 to 550 nm.

For the pentacene-doped PEDOT:PSS thin films, the UV-visible transmittance spectra are shown in Figure 1. As the amount of pentacene was increased, the UV-visible transmittance intensity slightly increased in the wavelength range of 300 ~ 800 nm. Therefore, the transmittance was dependent on the amount of pentacene doped in the PEDOT:PSS solution. Despite the increase in transparency of pentacene-doped PEDOT:PSS films, there is no relationship between transparency and conductivity.

**Figure 2:**
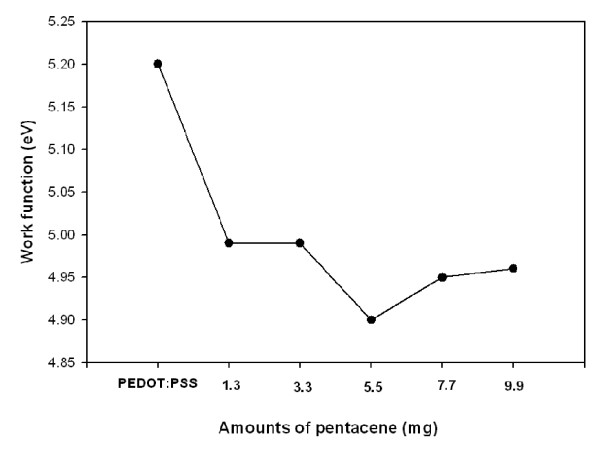
**Work function of pentacene-doped PEDOT:PSS films**.

**Figure 3:**
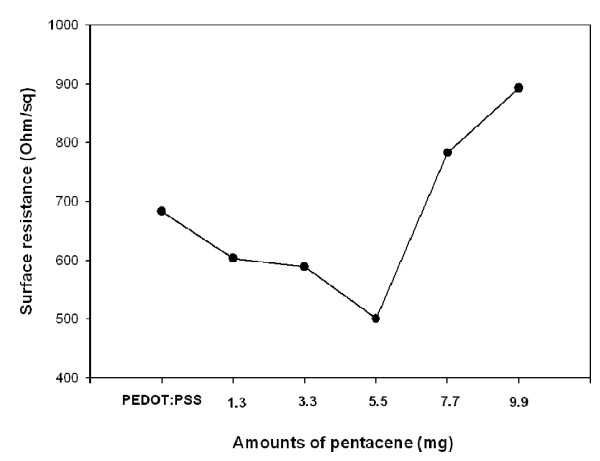
**Surface resistance of pentacene-doped PEDOT:PSS films**.

The work function variations and surface resistance of pentacene-doped PEDOT:PSS films are shown in Figures 2 and 3. The surface resistance was determined from the average value of measurements at multiple points on one sample in ambient condition. For a reliable analysis, the thickness of pristine PEDOT:PSS and pentacene-doped PEDOT:PSS films are fixed at about 40 nm. The work function and surface resistance decreased as the amounts of pentacene were increased in the PEDOT:PSS films. However, with pentacene amounts of 7.7 and 9.9 mg, the work function was slightly increased. The work function is correlative with the *V*_oc _value and hole-charge mobility to increase device efficiency [17]. The work function of the pristine PEDOT:PSS film was approximately 5.20 eV, and it decreases dramatically from 5.2 to 4.9 eV when it is doped with pentacene. The work function of PEDOT:PSS has been limited by charge collection because the work function of PEDOT:PSS film is higher than that of the HOMO level of pentacene. The bandgap of the pentacene-doped PEDOT:PSS film has been approached to the ITO substrate. Therefore, the amount of pentacene has been optimized to 5.5 mg, and the charge collection efficiency for the 5.5 mg of pentacene-doped film has been significantly increased; consequently, holes can easily move to the ITO substrate. By increasing the amount of pentacene in PEDOT:PSS films, a dramatic increase in the surface resistance is observed. With 7.7 and 9.9 mg of pentacene in PEDOT:PSS films, there were steep increases in the surface resistance, indicating that the conductivity of pentacene-doped PEDOT:PSS films significantly decreases as the pentacene doping amount exceeds 5.5 mg.

**Figure 4:**
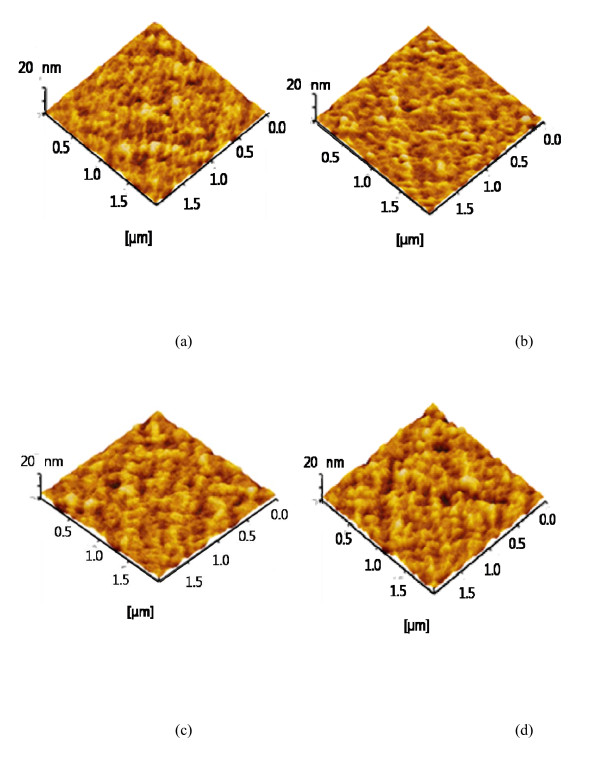
**AFM images of pentacene-doped PEDOT:PSS films**. Annealed at (**a**) 120°C, (**b**) 140°C, (**c**) 160°C, and (**d**) 180°C for 1 h.

Atomic force microscopy [AFM] images of pentacene-doped PEDOT:PSS films after annealing treatments are shown in Figure 4. After the amount of pentacene was optimized to 5.5 mg, the pentacene-doped PEDOT:PSS thin film was thermally annealed. As the annealing temperature was increased, the polymer aggregate or grain size also increased, and eventually, the continuous interfaces are formed, which improve conductivity through the interfaces of grains. As the annealing temperature was increased, the root-mean-square [RMS] surface roughness of pentacene-doped PEDOT:PSS films increased as well because the grain size has increased. For the pentacene-doped PEDOT:PSS annealed at 120°C for 1 h, a surface with an RMS roughness of 4.843 nm was observed. The pentacene-doped PEDOT:PSS films annealed at 140°C, 160°C, and 180°C show an RMS roughness of 5.267, 7.774, and 8.838 nm, respectively. Since the roughness is considered to be a signature of phase separation as well as grain formation in an active layer, the increase in the roughness of pentacene-doped PEDOT:PSS films leads to an improvement in the conductivity and charge mobility on their regions.

**Figure 5:**
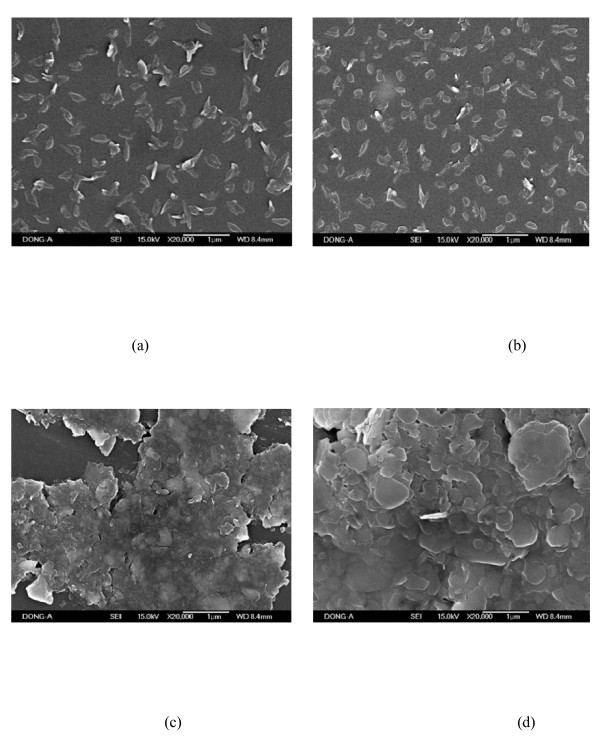
**SEM images of pentacene-doped PEDOT:PSS films**. Annealed at (**a**) 120°C, (**b**) 140°C, (**c**) 160°C, and (**d**) 180°C for 1 h.

At the lowest annealing temperature, the pentacene-doped PEDOT:PSS film shows uniformly dispersed small grains, indicating that crystalline density is high as shown in Figure 5. The low nucleation density leads to a large grain size at high temperature, thus leading to more grain boundaries [18]. As the annealing temperature increases, the grain surface also increases, leading to enhanced interfacial adhesion between buffer layer and active layer phases. It is observed in typical organic devices that the measured hole mobility increases along with the increase of the annealing temperature, starting to increase at a low temperature and saturating at a high temperature. The pentacene-doped PEDOT:PSS as a buffer layer exhibited annealing temperature dependence of charge mobility. Consequently, the pentacene-doped PEDOT:PSS film which is annealed at 180°C exhibits better molecular microstructure on the film surface and higher charge mobility.

**Figure 6:**
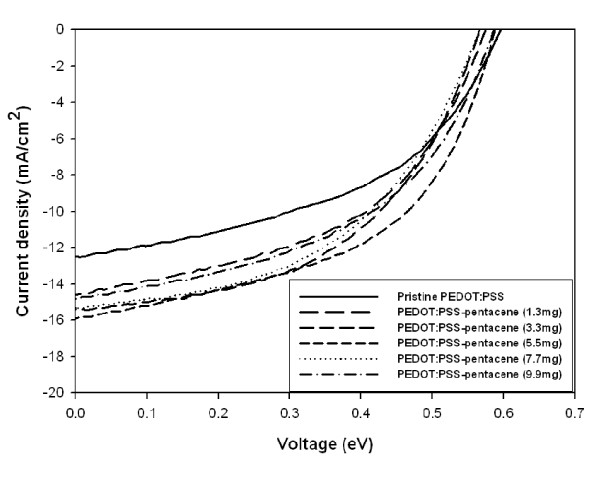
***J*-*V *characteristics of polymer photovoltaic devices using pentacene-doped PEDOT:PSS as a hole-conducting layer**.

The current density-voltage characteristics of polymer photovoltaic cells are shown in Figure 6. The polymer photovoltaic cells with the structure of ITO/pentacene-doped PEDOT:PSS (40 nm; 180°C) and photovoltaic cells with the structure of ITO/pentacene (40 nm; 180°C for 1 h)/P3HT:PCBM (2.0 wt.%; 1:0.9)/Al (100 nm) were fabricated. The device containing the PEDOT: PSS film has a *J*_sc _of 12.46, and the overall PCE of 3.74% was obtained for this device. For the device containing pentacene (5.5 mg)-doped PEDOT:PSS as a buffer layer, the *J*_sc _increases from 12.46 to 16.91 mA/cm^2^. Finally, the power conversion efficiency of 5.25% has been achieved. This improvement is attributed to an increase in the conductivity and work function resulting from pentacene doping into the PEDOT:PSS buffer layer. It is believed that the roughness of the pentacene-doped PEDOT:PSS film may induce the contact area between the buffer layer and the active layer. The hole-transporting ability is enhanced when increasing the conductive domains, therefore, leading to an improvement in *J*_sc_. However, its value was slightly decreased to 15.31 and 14.81 mA/cm^2 ^for 7.7 and 9.9 mg of pentacene doping, respectively. In this study, we demonstrated that a power conversion efficiency of 5.25%, by optimizing pentacene doping to 5.5 mg, has been achieved, and the annealing temperature of 180°C is preferred.

## Conclusions

In summary, the performance characteristics of polymer BHJ photovoltaic cells using pentacene-doped PEDOT:PSS as a buffer layer and a P3HT/PCBM-blended active layer have been investigated. By doping pentacene into PEDOT:PSS, the conductivity and carrier mobility of the buffer layer were improved. As the amount of pentacene was increased, the work function decreased. The bandgap of the pentacene-doped PEDOT:PSS film has been approached to the ITO substrate. The surface resistance decreased by pentacene doping in PEDOT:PSS films was also observed. In a morphological aspect, as the annealing temperature of pentacene-doped PEDOT:PSS thin films was increased, PEDOT:PSS formed aggregates or grains, which eventually improve the conductivity and hole-charge mobility. In this study, a power conversion efficiency of 5.25% has been achieved by doping pentacene into a PEDOT:PSS film.

## Abbreviations

AFM: atomic force microscopy; BHJ: bulk-heterojunction; HOMO: highest occupied molecular orbital; ITO: indium tin oxide; *J*_sc_: short circuit current; NMP: 1-methyl-2-pyrrolidinine; OLED: organic light-emitting diodes; PCBM: [6,6]-phenyl-C_61_-butyric acid methyl ester; PEDOT:PSS, poly(3,4-ethylenedioxythiophene:poly(4-styrenesulfonate); PTFE: polytetrafluoroethylene; P3HT: poly(3-hexylthiophene-2,5-diyl); RMS: root mean square; SEM: scanning electron microscopy; *V*_oc_: open circuit voltage.

## Competing interests

The authors declare that they have no competing interests.

## Authors' contributions

HK conceived the study, carried out the fabrication of photovoltaic cells, and drafted the manuscript. JL and SO estimated the photovoltaic cells and helped analyze the data. YC helped to develop the idea, guided the study, and drafted the manuscript. All authors read and approved the final manuscript.

## Authors' information

HK, JL, and SO are students of a Master's degree in the Chemical Engineering Department, Pusan National University, South Korea. YC is a professor in the Chemical Engineering Department, Pusan National University, South Korea.
